# Versican G1 domain enhances adenoviral-mediated transgene expression and can be modulated by inhibitors of the Janus kinase (JAK)/STAT and Src family kinase pathways

**DOI:** 10.1074/jbc.M116.773549

**Published:** 2017-07-06

**Authors:** Patricia Y. Akinfenwa, Wesley S. Bond, Cristhian J. Ildefonso, Mary Y. Hurwitz, Richard L. Hurwitz

**Affiliations:** From the ‡ Interdepartmental Program in Translational Biology and Molecular Medicine,; ¶ Texas Children's Cancer and Hematology Centers, and; ‖ Center for Cell and Gene Therapy and; the Departments of § Pediatrics,; ** Molecular and Cellular Biology, and; ‡‡ Ophthalmology Baylor College of Medicine, Houston, Texas 77030

**Keywords:** adenovirus, gene therapy, Janus kinase (JAK), STAT transcription factor, versican (VCAN)

## Abstract

To examine the biochemical influences that may contribute to the success of gene therapy for ocular disorders, the role of versican, a vitreous component, in adenoviral-mediated transgene expression was examined. Versican is a large chondroitin sulfate-containing, hyaluronic acid-binding proteoglycan present in the extracellular matrix and in ocular vitreous body. Y79 retinoblastoma cells and CD44-negative SK-N-DZ neuroblastoma cells transduced with adenoviral vectors in the presence of versican respond with an activation of transgene expression. Proteolysis of versican generates a hyaluronan-binding G1 domain. The addition of recombinant versican G1 to SK-N-DZ cells results in a similar activation of transgene expression, and treatment with dasatinib, an inhibitor of Src family kinases, also mimics the effects of versican. Enhancement is accompanied by an increase in signal transducer and activator of transcription 5 (STAT5) phosphorylation and is abrogated by treatment with C188-9, a STAT3/5 inhibitor, or with ruxolitinib, a Janus kinase 1/2 (JAK1/2) inhibitor. These data implicate versican G1 in enhancing adenoviral vector transgene expression in a hyaluronic acid-CD44 independent manner that is down-regulated by inhibitors of the JAK/STAT pathway and enhanced by inhibitors of the Src kinase pathway.

## Introduction

The presence of versican (VCAN)^5^ in the extracellular matrix (ECM) has been shown to influence cell phenotype, proliferation, migration, and adhesion (1). VCAN is a hyaluronic acid (HA)-binding proteoglycan component of the ECM and vitreous that has been shown to interact with CD44 in the presence or absence of HA (2). Five splice variants (V0–V4) of VCAN that are composed of different combinations of a G1 domain (containing an immunoglobulin-like module and two HA-binding link modules), alternatively spliced chondroitin sulfate-binding GAGα and GAGβ sequences, and a G3 domain (epidermal growth factor repeats, a lectin-like module, and a CRP-like module) have been described (3). A V5 isoform that predominantly contains the G1 domain may also exist (2). The larger variants V0 and V1 can be proteolyzed by a disintegrin-like and metalloprotease domain with thrombospondin type 1 (ADAMTS) protease and by matrix metalloprotease family members, releasing the amino-terminal G1 domain (also called versikine) and the carboxyl-terminal G3 domain (3). Through the interaction of these domains with extracellular receptors, intracellular signaling pathways that can influence metabolic and functional behaviors of the cells are activated. The G1 domain contains the HA-binding sites and promotes cell-matrix interactions by stabilizing ECM interactions such as between HA, link protein, and fibrillin microfibrils (4, 5). Studies have implicated G1 overexpression in mediating cell adhesion and proliferation (6, 7), but its function and signaling mechanisms remain areas of active research. The carboxyl-terminal G3 domain contains epidermal growth factor-like elements and a lectin-like domain and has been shown to play a role in cancer by regulating cell adhesion, metastasis, and apoptosis (8–11).

In addition to its function in the extracellular matrix, VCAN is the major proteoglycan in ocular vitreous humor that also contains high concentrations of collagen and HA. Both preclinical studies and clinical trials using viral vectors to deliver transgenes for the treatment of retinoblastoma have demonstrated more therapeutic potential than non-ocular applications using the same vectors (12, 13). The immunologic and the biochemical environments of the eye have been implicated in this increased therapeutic potential (14, 15). The immune privilege associated with the eye, anterior chamber-associated immune deviation, protects the intraocular structures and limits the systemic immune response to the proteins produced by the transgenes and to the viral vectors themselves (16, 17). *In vitro* examination of vitreous components have implicated HA and interactions with its receptor CD44 with increased expression of transgenes delivered by adenoviral vectors. However, digesting vitreous with hyaluronidase or antagonizing the HA-CD44 interaction resulted in only a partial reduction in enhancement, suggesting an HA-CD44-independent mechanism that remains unexplained (15).

In this study, we investigate the VCAN G1 domain and the VCAN-activated signaling pathways by measuring the expression of luciferase reporter gene delivered by an adenoviral vector to two different cell lines. Y79 retinoblastoma cells represent the ocular tumors targeted by the first trial of gene therapy in the eye (13). SK-N-DZ neuroblastoma cells that are CD44-negative and do not bind HA (18) were used to isolate the mechanisms being investigated to HA-CD44 independent steps. Understanding the signaling mechanisms mediated by versican can provide further insight into the molecular mechanisms involved in the exchange of information between the cells and the extracellular matrix as well as how an adenovirus manipulates normal cellular functions for its own replication. This information will also provide the basis for the design of more effective antiviral therapies and for the design of viral-mediated therapies for a wide range of genetic and oncogenic disorders and diseases.

## Results

### Versican activates the expression of adenoviral vector transgenes in the absence of CD44

**Figure 1.**
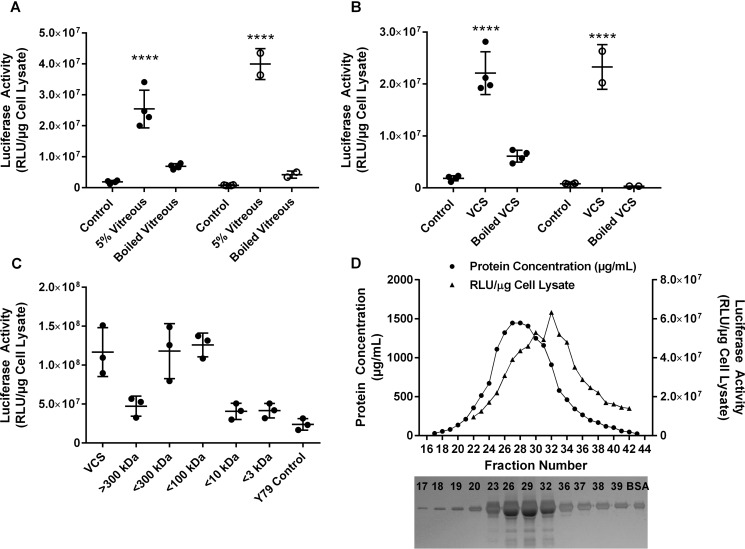
**Versican activated the expression of adenoviral vector transgenes.** *A*, Y79 (●) or SK-N-DZ (○) cells were transduced with Ad5/CMV-Luc (multiplicity of infection 250 or 50 pfu/cell, respectively) in the presence of unheated 5% vitreous or vitreous preheated at 95°C for 5 min. Twenty-four hours post-transduction, luciferase activity was determined as a measure of transgene expression. Incubation of either cell line with 5% vitreous resulted in a significant increase (*p* < 0.0001) of transgene expression. Heating vitreous prevented the vitreous-mediated increase in luciferase activity. *B*, Y79 (●) or SK-N-DZ (○) cells were transduced with Ad5/CMV-Luc (multiplicity of infection 250 or 50 pfu/cell, respectively) in the presence of unheated 5% VCS or VCS preheated at 95°C for 5 min. Twenty-four hours post-transduction, luciferase activity was determined as a measure of transgene expression. Incubation of either cell line with 5% VCS resulted in a significant increase (*p* < 0.0001) of transgene expression. Heating VCS prevented the vitreous-mediated increase in luciferase activity. *C*, to determine the size range of activating components, VCS was subjected to membrane filtration. Centrifugal filters with molecular mass cut offs of 300 kDa, 100 kDa, 10 kDa, and 3 kDa were used sequentially with each filtrate applied to the next smaller molecular mass cut off filter. Luciferase assays of each filtrate indicated that the active component of VCS was between 300 kDa and 10 kDa, suggesting that the active VCS protein does not contain chondroitin sulfate moieties and may be a proteolytic fragment. *D*, Sepharose CL 4B gel filtration chromatography was used to confirm the presence of low molecular weight active species. The enhancing activity eluted in fractions behind bovine serum albumin, indicating that an active species is present in the VCS that could correspond to a proteolytic fragment of versican. A silver-stained polyacrylamide gel and amino acid sequencing confirmed the BSA protein alignment with the protein peak that eluted just before the active component. Experiments were repeated at least three times with internal replicates of two to four. *RLU*, relative luminescence units. ****, *p* < 0.0001.

Incubation of Y79 retinoblastoma cells with ocular vitreous humor enhances adenoviral mediated transgene expression (15, 19). This result was independent of viral internalization and was the result of increased viral transcription. CD-44-negative, neuroblastoma-derived SK-N-DZ cells engineered to express CD-44 show that the interaction between HA and CD44 was partially responsible for the adenoviral-mediated enhancement effect. However, much of the enhancement was independent of CD44 expression. Incubating Y79- or CD44-negative SK-N-DZ cells with an adenoviral vector delivering the luciferase gene (Ad5/CMV-Luc) in the presence of vitreous (5% v/v) that had been heated to 95 °C for 5 min did not result in an increase in luciferase activity, indicating that a heat-labile component of vitreous was at least in part responsible for the increase in transgene expression (Fig. 1*A*). Therefore, there appears to be an additional, hyaluronan-independent mechanism that can activate adenoviral-mediated transgene expression. VCAN is a hyaluronan-binding, chondroitin sulfate-containing proteoglycan known to be a component of vitreous. VCAN, which can interact with CD44 through its bound hyaluronan, was hypothesized to be a protein that might be responsible for both CD44-dependent and -independent mechanisms that influence adenoviral-mediated transgene expression. VCAN is secreted into the culture media by ACHN renal adenocarcinoma cells (20). Incubation of Y79 or SK-N-DZ cells with the versican-containing culture medium (VCS) from ACHN cells followed by transduction with Ad5/CMV-Luc resulted in increased luciferase activity similar to that achieved with vitreous. Thermal lability of the VCS component was verified by heating the conditioned media at 95 °C for 5 min before inclusion in the transduction assays (Fig. 1*B*).

Versican, with its associated glycosaminoglycans, has been purified from ACHN VCS and shown to have a molecular mass of 1600 kDa (20). To determine whether the component of VCS responsible for the enhancement of viral-mediated transgene expression is the large, fully glycosylated versican or either the core protein alone or a proteolytic fragment, two approaches were used, ultrafiltration and Sepharose CL-4B gel filtration chromatography. First, VCS was subjected to sequential membrane filtration using polyethersulfone (PES) filters with molecular mass cut-offs of 300 kDa, 100 kDa, 10 kDa, and 3 kDa (Sartorius Stedim, Bohemia, NY). Fractions were assayed for their ability to enhance transgene expression in Y79 cells transduced with Ad5/CMV-Luc. The first filter with a molecular mass cut off of 300 kDa that would retain the large, fully glycosylated VCAN allowed the enhancing activity to flow through the filter. The filtrate was then passed through a filter with a molecular mass cut off of 100 kDa and again enhancing activity passed through the filter. Very little if any activity passed through the 10-kDa filter, suggesting that an active species has a molecular mass between 10 kDa and 100 kDa (Fig. 1*C*).

VCS was also subjected to Sepharose CL-4B size-exclusion chromatography. In our preparation, the activity was found in fractions with slightly smaller molecular mass than bovine serum albumin (Fig. 1*D*). Although these experiments do not exclude a role for fully glycosylated versican, these two experiments together suggest that there is an active species that does not contain multiple glycosylation attachments and that it could be one of the naturally occurring proteolytic fragments of VCAN.

### Characterization of VCAN G1 domain activation of adenoviral transgene expression

**Figure 2.**
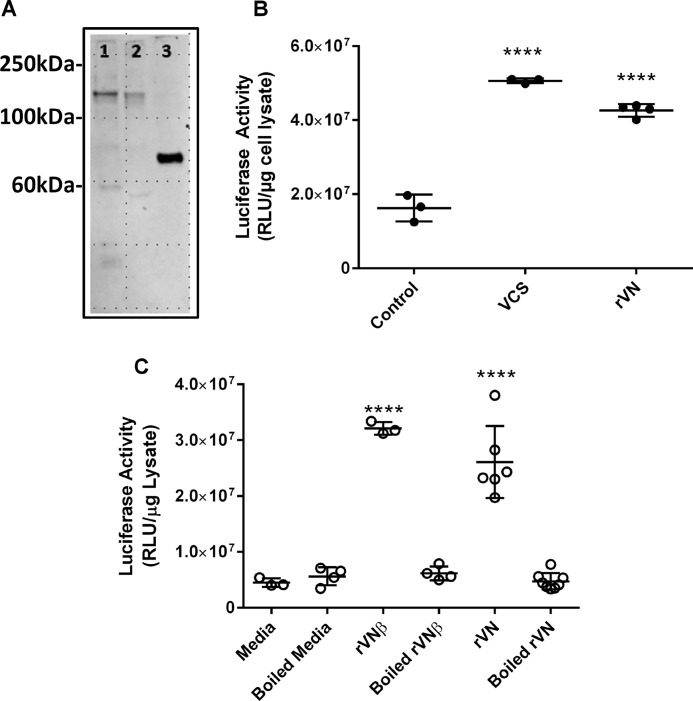
**Characterization of VCAN G1 domain activation of adenoviral transgene expression.** *A*, vitreous, VCS, or rVNβ samples were electrophoresed through 4–12% gradient bis-tris acrylamide gels using non-reducing conditions. The proteins were transferred to a polyvinylidene difluoride membrane, and the blot was probed with 12C5 (1:250 dilution), an antibody to the G1 domain of versican. 12C5 recognized protein bands at 150 kDa that correspond to the predicted mass of core versican protein in both vitreous (*lane 1*) and VCS (*lane 2*). The band at 70 kDa in *lane 3* corresponds to the reported position for rVNβ (22, 38). *RLU*, relative luminescence units. *B*, Y79 cells were incubated with VCS or rVN and then transduced with Ad5/CMV-Luc. VCS and rVN both significantly increased transgene expression as measured by luciferase activity when compared with mock transfected controls. ****, *p* < 0.0001. *C*, SK-N-DZ cells were incubated with rVNβ or rVN or with rVNβ or rVN that had been preheated at 95°C for 5 min. The cells were then transduced with Ad5/CMV-Luc, and luciferase activity was determined. Both rVNβ and rVN significantly increased luciferase activity (*p* < 0.0001); however, preheating blocked the response. Experiments were repeated at least three times with internal replicates of three or more. ****, *p* < 0.0001.

To examine the role of the G1 domain in the activation of adenoviral transgene expression, two recombinant versican G1 domain constructs were obtained from Dr. Zenzo Isogai. The recombinant versican polypeptide β (rVNβ) (versikine) and rVN constructs, respectively, correspond to Leu^21^–Glu^441^ and Leu^21^–Arg^348^ of the VCAN V1 isoform and were engineered to contain a SPARC signal peptide (AAs 1–20) to facilitate peptide secretion (21). The constructs both contain the immunoglobulin (Ig)-like domain (AAs 36–150) and two link module domains (1, AAs 151–244; 2, AAs 248–347), each of which contains an HA-binding region (1, Arg^160^–Tyr^161^) and (2, Pro^258^–Ser^259^). To determine whether G1 plays a role in enhancement, the constructs were transiently expressed in HepG2 cells. After the cells were incubated for 7–10 days, the media containing secreted rVCANs were collected and concentrated. A Western blot of vitreous, VCS, and rVNβ was prepared and developed using a monoclonal antibody 12C5 that recognizes the VCAN G1 domain (Fig. 2*A*). Bands corresponding to the VCAN core protein with a molecular mass of ∼150 kDa were detected in both the vitreous and VCS lanes. A band in the rVNβ lane was detected at ∼70 kDa as predicted for versikine (22). To determine whether the smaller rVN fragments can enhance transgene expression, Y79 cells were incubated with VCS or rVN and transduced with Ad5/CMV-Luc (Fig. 2*B*), and SK-N-DZ cells were incubated with rVNβ or rVN with or without preheating and transduced with Ad5/CMV-Luc (Fig. 2*C*). Luciferase activity was significantly increased when compared with medium from mock-transfected controls, suggesting that the G1 domain is sufficient to enhance adenoviral-mediated gene expression.

### Src kinase inhibition activates adenoviral transgene expression

**Figure 3.**
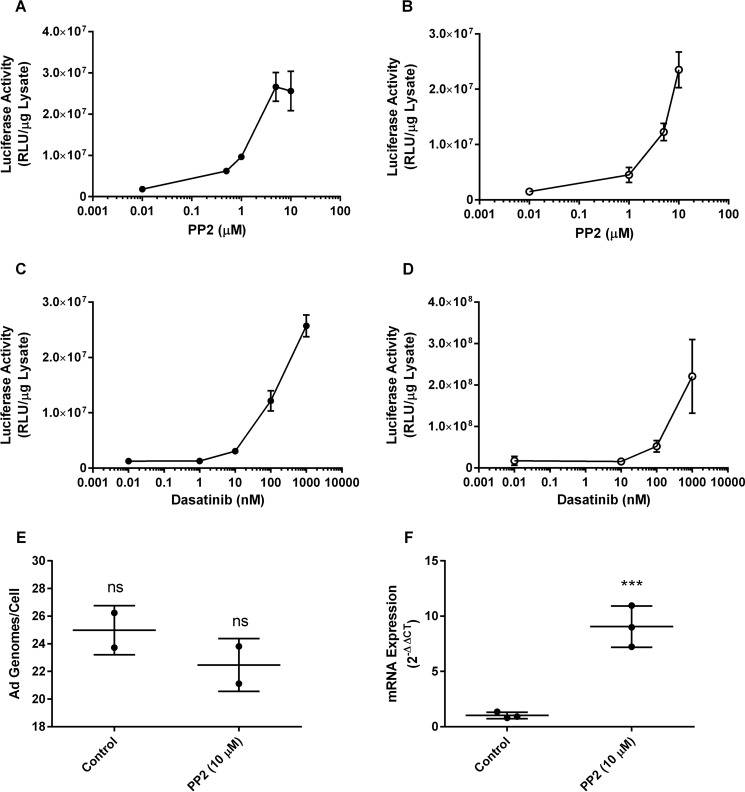
**Src kinase inhibition activated adenoviral transgene expression.** The Src kinase inhibitor PP2 was used to generate dose-response curves in Y79 cells (*A*) or SK-N-DZ cells (*B*) that were then transduced with Ad5/CMV-Luc. Luciferase activity, determined as a measure of transgene expression, increased in a similar dose-dependent manner in both cell lines. Similar results were obtained when dose-response curves were generated using an alternate Src kinase inhibitor dasatinib using Y79 (*C*) or SK-N-DZ (*D*) cells that were then transduced with Ad5/CMV-Luc. All curves were generated with internal replicates of three. Y79 cells were transduced with Ad vector with a chimeric serotype 35 fiber protein delivering an IL-12 transgene (Ad5F35/CMV-IL-12) and treated with 5 μm PP2 for 18 h, after which total DNA and RNA were isolated. Quantitative PCR shows no increase in the Ad genome copy number internalized by the cells (*E*) but a significant increase in IL-12 mRNA levels (*F*). PP2 did not affect viral internalization but significantly increased mRNA expression (***, *p* < 0.001). *RLU*, relative luminescence units. *ns*, not significant.

VCAN has been shown to promote neuronal differentiation and neurite outgrowth that is blocked by inhibition of Src family kinases (23). The Src family kinase activities in the cancer cell lines used in these studies are modulated in response to cell-cell contact or other differentiation stimulators (24, 25). We, therefore, hypothesized that Src kinase activity may play a role in regulating viral gene expression. To determine whether Src kinase plays a role in the expression of adenoviral transgenes, Y79 cells (Fig. 3, *A* and *C*) or SK-N-DZ cells (Fig. 3, *B* and *D*) were transduced with Ad5/CMV-Luc in the presence of increasing doses of the Src kinase small-molecule inhibitors PP2 (Fig. 3, *A* and *B*) or dasatinib (Fig. 3, *C* and *D*). Incubation with either inhibitor resulted in increased luciferase activity in a dose-dependent manner. The increase in activity was not due to increased virus internalization (Fig. 3*E*) but was associated with an increase in RNA transcription (Fig. 3*F*). That vitreous, VCS, and Src kinase inhibition converge on one or more pathways to activate adenoviral transgene expression is possible.

### Janus kinase (JAK) signaling and enhancement of adenoviral transgene expression

The JAKs form a family of receptor-tyrosine kinases involved in intracellular signaling cascades through their association with cell-surface receptors for ligands such as IL-6, interferon, and growth hormone (26–28). Their activation has been shown to regulate tumor cell proliferation, survival, and invasion by mediating the tyrosine phosphorylation and activation of signal transducer and activator of transcription (STAT) family members (29). JAK, activated itself by phosphorylation, initiates recruitment of STAT molecules to the JAK/JAK–receptor complex. A recruited STAT molecule is phosphorylated by JAK, initiating its homo- or heterodimerization with a second pSTAT family member. The newly formed dimer undergoes nuclear translocation where it regulates gene transcription.

**Figure 4.**
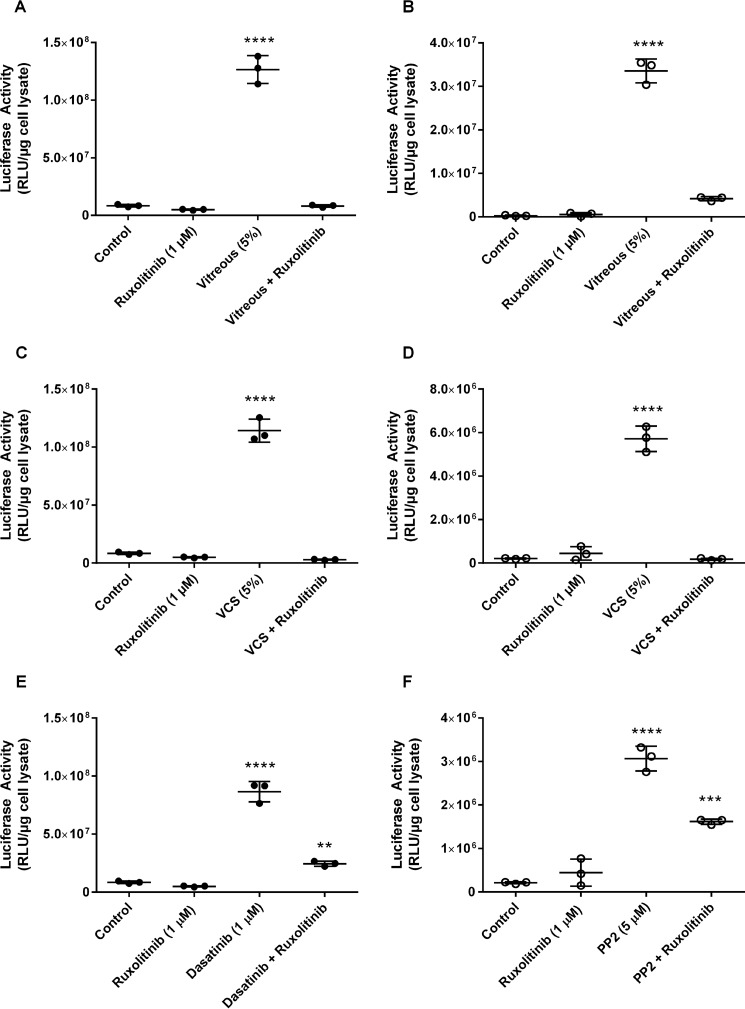
**JAK signaling and enhancement of adenoviral transgene expression.** Y79 or SK-N-DZ cells were incubated with vitreous (5%), VCS (5%), dasatinib (1 μm), or PP2 (5 μm) in the presence or absence of the JAK1/2 inhibitor ruxolitinib (1 μm). After transduction with Ad5/CMV-Luc, luciferase activity was assayed as a measure of transgene expression. Activation of transgene expression was significant (****, *p* < 0.0001) when compared with the controls. Ruxolitinib inhibited the activation by vitreous, VCS, or src kinase inhibitors. *A*, Y79 cells activated with vitreous (5%). *B*, SK-N-DZ cells activated with vitreous (5%). *C*, Y79 cells activated with VCS (5%). *D*, SK-N-DZ cells activated with VCS (5%). *E*, Y79 cells activated with dasatinib (1 μm). **, *p* < 0.01. *F*, SK-N-DZ cells activated with PP2 (5 μm). Experiments were repeated at least three times with internal replicates of three or more. *RLU*, relative luminescence units. ***, *p* < 0.001.

To determine which of these pathways was important in the regulation of adenoviral transgene expression, Y79 cells were treated with vitreous, VCS, or a Src kinase inhibitor and transduced with Ad5/CMV-Luc in the presence or absence of a small-molecule inhibitor of either the mTOR- or JAK-signaling pathways, both of which have been shown to regulate transcription downstream of Src kinase. To test the importance of the mTOR pathway, Y79 cells were treated with vitreous (5%) and transduced with Ad5/CMV-Luc in the presence or absence of the mTOR inhibitor 100 nm everolimus (30). Inhibition of mTOR did not alter the vitreous-mediated enhancement of transgene expression (data not shown). To determine the importance of JAK signaling in the enhanced expression of genes delivered by adenoviral vectors, Y79 or SK-N-DZ cells were treated with vitreous (5%), VCS (5%), or one of the Src kinase inhibitors PP2 (5 μm) or dasatinib (1 μm) (31) and transduced with Ad5/CMV-Luc in the presence or absence of 1 μm JAK1/2 inhibitor ruxolitinib (32) followed by analysis of luciferase activity. Although JAK inhibition had no apparent effect on baseline transgene expression, incubation in the presence of the JAK inhibitor resulted in an inhibition of vitreous, VCS, dasatanib, and PP2-mediated enhancement of transgene expression (Fig. 4, *A–F*). This suggests that each of the activators converge upon a single pathway in transcriptional regulation.

### Enhancers of adenoviral transgene expression increase STAT5 phosphorylation

**Figure 5.**
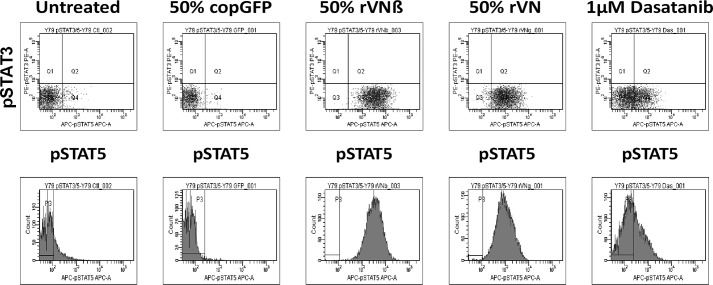
**Enhancers of adenoviral transgene expression increased stat5 phosphorylation.** The effect of versican G1 domain treatment on JAK2 activation was measured by treating Y79 cells with control supernatant (media or copGFP), supernatant expressing rVNβ or rVN, or 1 μm dasatanib for 1 h. Cells were fixed in paraformaldehyde, permeabilized using 100% methanol, and probed with PE- or FITC-conjugated antibodies for pSTAT3 or pSTAT5, respectively and analyzed by flow cytometry. Flow cytometry analysis revealed that a 1 h treatment with rVNβ, rVN, or the Src kinase inhibitor dasatinib resulted in an increase in STAT5 but not STAT3 phosphorylation.

JAK2 has been shown to phosphorylate STAT3 and STAT5; thus STAT3 or STAT5 phosphorylation is commonly used to infer JAK2 activation. To determine if JAK2 signaling plays a role in the versican-mediated activation of adenoviral transgene expression, the phosphorylation status of STAT3 (pSTAT3) and of STAT5 (pSTAT5) was assessed. The effect of versican G1 domain treatment on JAK2 activation was measured by treating Y79 cells with control supernatant (media from untreated HepG2 cells or media from HepG2 cells transfected with a control plasmid expressing GFP (copGFP)) or supernatant expressing rVNβ, rVN, or 1 μm dasatanib for 1 h. Cells were fixed in paraformaldehyde, permeabilized using 100% methanol, and probed with PE- or allophycocyanin (APC)-conjugated antibodies for pSTAT3 or pSTAT5, respectively and analyzed by flow cytometry. Flow cytometry analysis revealed that a 1-h treatment with rVN resulted in an increase in STAT5 but not STAT3 phosphorylation (Fig. 5). These results suggest that JAK2 is activated upon treatment with the G1 domain of versican.

### Inhibition of the JAK/STAT pathway inhibits the enhancement of adenoviral transgene expression

**Figure 6.**
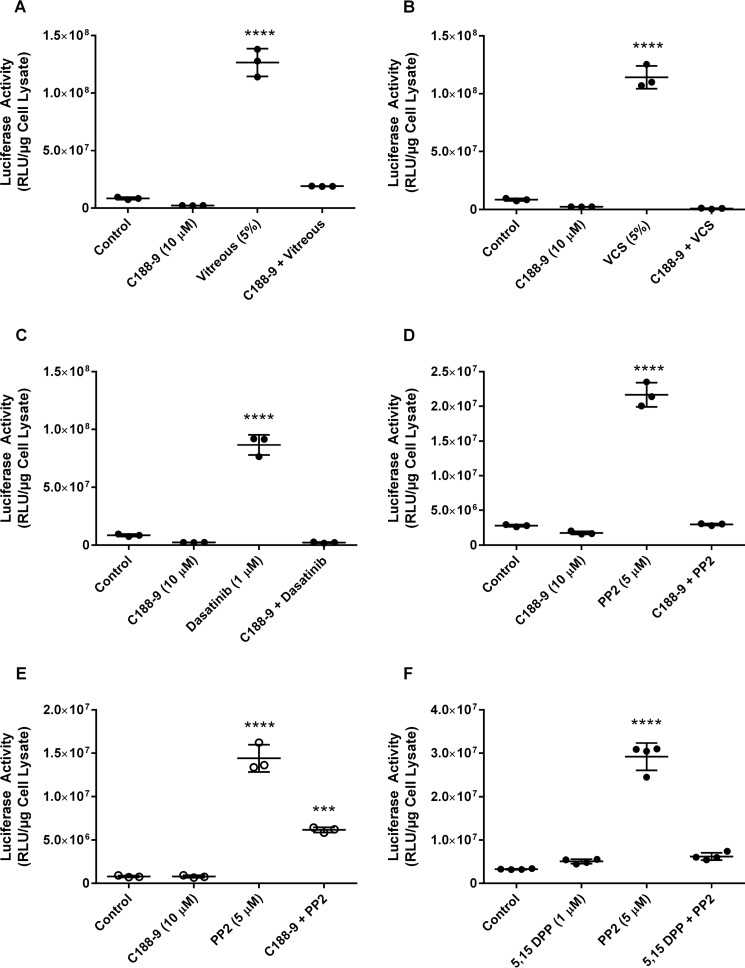
**Inhibition of the JAK/STAT pathway inhibited the enhancement of adenoviral transgene expression.** Y79 (●) or SK-N-DZ (○) cells were transduced with Ad5/CMV-Luc in the presence of vitreous (5%), VCS (5%), dasatinib (1 μm), or PP2 (5 μm) in the presence or absence of the STAT3/5 inhibitors C188-9 (10 μm) or 5,15-DPP (1 μm). Luciferase activity was assayed as a measure of transgene expression. Significant activation (*p* < 0.0001) was observed regardless of the cell line or transgene expression activator examined. The activation was reduced to baseline by inhibition of STAT. *A*, Y79 cells activated with vitreous (5%) and treated with C188-9 (10 μm). ****, *p* < 0.0001. *RLU*, relative luminescence units. *B*, Y79 cells activated with VCS (5%) and treated with C188-9 (10 μm). *C*, Y79 cells activated with dasatinib (1 μm) and treated with C188-9 (10 μm). *D*, Y79 cells activated with PP2 (5 μm) and treated with C188-9 (10 μm). *E*, SK-N-DZ cells activated with PP2 (5 μm) and treated with C188-9 (10 μm). ***, *p* < 0.001. *F*, Y79 cells activated with PP2 (5 μm) and treated with 5,15-DPP (1 μm). Experiments were repeated at least twice with internal replicates of three or more.

Because the inhibition of JAK1/2 mitigated the enhancement of transgene expression (Fig. 4) and STAT5 phosphorylation increased upon enhancer treatment (Fig. 5), it is possible that activation of the JAK/STAT pathway could play a role in mediating the increased expression of adenoviral transgenes after treatment with vitreous, versican, or Src kinase inhibitors. To further confirm a role for STAT5 in this enhancement, STAT3/5 inhibitors C188-9 (33) or 5,15-DPP (34) were employed. Y79 or SK-N-DZ cells were transduced with Ad5/CMV-Luc in the presence of vitreous (5%), VCS (5%), dasatinib (1 μm), or PP2 (5 μm) in the presence or absence of C188-9 (10 μm) or 5,15-DPP (1 μm), and luciferase activity was measured (Fig. 6). Although incubation with the STAT inhibitors resulted in little change in baseline transgene expression in the absence of vitreous, VCS, or Src inhibition, luciferase activity was mitigated after treatment with the STAT3/5 inhibitors in the presence of vitreous, VCS, or Src inhibitors. Taken together, these results suggest the enhancement of adenoviral vector-mediated transgene expression occurs by signaling through the JAK/STAT pathway.

## Discussion

The results of this study uncovered that the heat labile, CD44-independent vitreous-mediated enhancement of adenoviral vector transgene expression is mediated at least in part by the G1 domain of versican. Chaudhuri *et al.* (19) and Ildefonso *et al.* (15) previously reported that ocular vitreous or the vitreous component hyaluronan could increase adenoviral-mediated transgene expression in transduced cells. This enhancement was the result of increased transgene expression and not viral internalization. However, hyaluronan could not alone explain the increase in transgene expression observed. There appeared to be a potentiating factor in vitreous as far lower concentrations of hyaluronan in vitreous could achieve the same activation of gene expression observed than when hyaluronan alone was used. Vitreous could also activate adenoviral-mediated gene expression when the SK-N-DZ cell line was used, a cell line known not to bind hyaluronan or contain hyaluronan-binding receptors (18). In this report, we demonstrate that boiling vitreous prevented enhancement of adenoviral-mediated transgene expression. Because hyaluronan is heat stable, an additional factor must be involved. We hypothesized that versican, a proteoglycan contained in vitreous, could be a component that can enhance adenoviral-mediated transgene expression in the presence or in the absence of hyaluronan-binding receptors. Human ACHN renal carcinoma cells secrete versican. Incubation of SK-N-DZ cells with the media supernatant of ACHN cultures mimics the effect of vitreous. The supernatants collected from other cell lines including HEK293 and HepG2 that were used as controls could not achieve this effect. The factor in the culture media from ACHN cells was heat-labile. Using both membrane filtration and size-exclusion chromatography, the active size of this factor was determined to be approximately the size of bovine albumin, whereas the molecular mass of versican including its glycan moieties approaches 2 million daltons. Versican can be metabolized by cellular proteases to release its G1 and G3 domains (35). The G1 domain contains the two hyaluronan-binding sites. Although the versican G3 domain has been widely implicated in cancer cell proliferation and metastasis, to date most reports about the G1 domain have been related to its roles in maintaining structural integrity or its interaction with other extracellular matrix molecules like hyaluronan. With the exception of a few studies implicating the G1 domain in increased cell growth and decreased adhesion (6, 7), the functional effects of the G1 domain remained unexplained. Western blot analysis using a monoclonal antibody that recognizes the G1 domain shows that the G1 domain was present in vitreous and the ACHN VCS preparation. Plasmids that express either versikine or the G1 domain were obtained. Supernatants from transfected HepG2 cell cultures that had been transfected with the plasmid could specifically enhance adenoviral-mediated transgene expression. This enhancement was found in CD44-negative SK-N-DZ cells, thus showing that the enhancement occurred in a hyaluronan-independent manner.

Downstream intracellular signaling pathways were investigated. Many of the cell lines tested *in vitro* have been shown to overexpress c-Src. For example, treating HepG2 hepatocellular carcinoma cells, a cell line that overexpresses c-Src, with vitreous or versican enhanced transgene expression. Conversely, vitreous or versican treatment of COS-7 cells, shown to have little c-Src expression (36), failed to activate transgene expression (data not shown). Results in this study revealed that small molecule inhibitors of c-Src enhanced adenoviral-mediated transgene expression, suggesting that the pathway mediating enhancement is sensitive to Src kinase expression and that c-Src may serve as a negative regulator of viral gene expression.

Treatment of cells with inhibitors of the JAK pathway blocked the enhancement of adenoviral-mediated gene expression when enhanced by vitreous, versican, or c-src inhibition. Incubation of the versican G1 domain with target cells resulted in an increase in STAT5 phosphorylation. Inhibitors of STAT phosphorylation and dimerization also blocked the enhancement of adenoviral-mediated transgene expression. We previously reported that hyaluronan CD44 interaction also enhanced viral-mediated transgene expression through metalloproteinase and γ-secretase metabolism of CD44 and release of its intracellular cytoplasmic domain (15). This peptide has been reported to interact with STAT and results in stabilizing its transcription activator activity (37). Whether this mechanism could explain how hyaluronan is responsible for boosting transgene expression in hyaluronan-binding cells remains to be explored. The JAK/STAT pathway is responsible for cytokine production that confers immunity to viral infection. One can only speculate whether this pathway evolved as a means of cellular counteraction to adenovirus infection or as the result of adenovirus evolving to counteract our immune system.

Versican G1 serves as a key component of the extracellular matrix, regulating interactions between multiple extracellular matrix members including HA and CD44. This report describes a role for the G1 domain that is independent of hyaluronan in enhancing adenoviral-mediated gene expression. The molecular details of the cellular pathway and its role in normal cellular physiology remain to be elucidated. The manipulation of c-Src, JAK, and STAT pathways with small molecular weight inhibitors could be used to improve gene therapy strategies using these vectors and possibly control adenoviral infections.

## Experimental procedures

### Cell cultures

Cell lines were propagated as follows: Y79 (ATCC®HTB-18^TM^) and COS7 (ATCC®CRL-1651^TM^) cells were cultured in DMEM (Mediatech, Manassas, VA) supplemented with 5% fetal bovine serum (FBS) (Benchmark, Gemini Bio-Products, Sacramento, CA); SK-N-DZ (ATCC®CRL-2149^TM^) cells were cultured in DMEM supplemented with 5% FBS and nonessential amino acids (Mediatech); ACHN (ATCC®CRL-1611^TM^) and HepG2 (ATCC®HB-8065^TM^) cells were cultured in MEM (Mediatech) supplemented with or without 5% FBS. All cultures were supplemented with 1% penicillin and streptomycin (Mediatech) and maintained in humidified conditions in 5% CO_2_ at 37 °C.

### Antibodies and chemicals

Versican monoclonal antibody (12C5) recognizing the G1 hyaluronan-binding domain of human versican was purchased from Developmental Studies Hybridoma Bank (Iowa University), and VCAN polyclonal antibody (ab19345) recognizing the ADAMTS-1/4 cleavage site (aa 436–441) was purchased from Abcam (San Francisco). The flow cytometry antibodies to pSTAT3 (Tyr(P)-705) and to pSTAT5 (Tyr(P)-694), conjugated to PE and to Alexa Fluor 647, respectively, were purchased from BD Biosciences. The Src family kinase inhibitor PP2 was purchased from EMD Biosciences (San Diego, CA). The Src family kinase inhibitor dasatinib and the JAK inhibitor ruxolitinib (INCB018424) were purchased from Selleck Chemicals (Houston, TX). STAT3/5 inhibitor C188-9 was obtained from Dr. Michelle Redell (Baylor College of Medicine), and the STAT3/5 inhibitor 5,15-DPP was purchased from Abcam (San Francisco, CA). Puromycin dihydrochloride was purchased from Mediatech. Doxycycline was purchased from Clontech (Mountain View, CA).

### Adenoviral vector

The first generation adenoviral vector serotype 5 delivering a luciferase (Luc) reporter gene under the control of the CMV promoter (Ad5/CMV-Luc) was prepared and expanded by the Vector Development Laboratory at Baylor College of Medicine.

### VCS

The renal adenocarcinoma cell line ACHN secretes VCAN V1 splice variant into its culture supernatant (20). ACHN cells (15 × 10^6^) were cultured for 7–10 days, and the medium was harvested and concentrated at 4 °C (20).

### Expression of rVN

Recombinant DNA constructs containing sequences that code for the versican G1 globular domain were kind gifts from Dr. Zenzo Isogai (National Center for Geriatrics and Gerontology, Morioka-cho, Obu, Aichi, Japan). The rVNβ construct encodes a polypeptide (versikine) corresponding to Leu^21^–Glu^441^ (5), and rVN construct encodes a polypeptide corresponding to the region Leu^21^–Arg^348^ of human versican V1 (21). The recombinant sequences were subcloned into pCEP/γ2III4, which contains a leader sequence for the BM40/SPARC (secreted protein, acidic and rich in cysteine) signal peptide to facilitate peptide secretion (5). The constructs were expanded and evaluated after transient transfection into HepG2 cells (2 × 10^6^ cells/10-cm dish) using the Lipofectamine 2000 reagent (Invitrogen). Parallel transfection of pCDH-CMV-MCS-EF1-copGFP facilitated verification of transgene expression as determined by fluorescence visualization. Cells expressing the rVN were incubated for 7–10 days to allow for secretion of the peptides into the growth media. Conditioned media was concentrated at least 10-fold using Concentrator Powder (G Biosciences) and Spectra/Por dialysis membrane (Spectrum Medical Industries, 6–8-kDa molecular mass cut off). The amino acid sequences of the expressed versican fragments were confirmed by Lone Star Labs.

### Preparation of vitreous

Each step of the vitreous extraction procedure was performed on ice or at 4 °C unless otherwise noted. Vitreous from frozen bovine eyes obtained from a local abattoir was isolated and prepared as previously described (15).

### Luciferase assay

Luciferase activity was used as a measurement of transgene expression. Recombinant luciferase enzyme (Promega, Madison, WI) was used to determine the linear range of a Triathler Luminometer, and all assays were adjusted to fall within this range. Cells were lysed using reporter lysis buffer (Promega) and assayed using a luciferase assay kit (Promega) according to the manufacturer's protocol. Luciferase activity is reported as relative luminescence per μg of total lysate protein determined by Bradford assays (Bio-Rad).

### SDS-PAGE and Western blotting

Twenty-microgram samples of cell lysate were denatured in non-reducing conditions using 4× NuPAGE sample buffer (Invitrogen) at 70 °C for 10 min before loading onto 4–12% gradient gels in preparation for SDS-PAGE. After separation, proteins were silver-stained using FASTsilver™ (Calbiochem) or transfer to polyvinylidene difluoride membranes (GE Healthcare) for Western analysis per the manufacturers' instructions.

### Statistical analysis

Graph Pad Prism 6 was utilized to analyze quantitative data. For comparison of more than two averages, a one-way analysis of variance test was performed followed by Tukey analysis to detect significant differences between all groups or between a control group and experimental groups, respectively. Experiments were repeated at least three times with experimental replicates of at least three. Most experiments were performed independently by two or more individuals in the laboratory, and representative results from a single experiment are shown. Statistical significance was assumed at *p* ≤ 0.05. Symbols denoting levels of statistical significance are: *, *p* < 0.0.05; **, *p* < 0.01; ***, *p* < 0.001; ****, *p* < 0.0001.

## Author contributions

R. L. H. and M. Y. H. oversaw the project, procured the funding, and verified all data analyses. C. J. I. and W. S. B. conceived and designed the studies related to VCAN. W. S. B. and P. Y. A. designed and performed the studies related to the JAK/STAT pathway. P. Y. A. performed the studies related to the recombinant VCAN domains. P. Y. A., R. L. H., and M. Y. H. wrote the paper. All authors approved the final version of the manuscript.

## Footnotes

5 The abbreviations used are:

VCAN: versican
VCS: versican-containing supernatant
JAK: Janus kinase
STAT: signal transducer and activator of transcription
ECM: extracellular matrix
HA: hyaluronic acid
ADAMTS: a disintegrin-like and metalloprotease domain with thrombospondin type 1
rVN: recombinant versican polypeptide
copGFP: control plasmid expressing GFP
PE: phosphatidylethanolamine
bis-tris: 2-[bis(2-hydroxyethyl)amino]-2-(hydroxymethyl)propane-1,3-diol
Ad: adenovirus
AA: amino acids
5,15-DPP: 5,15-diphenylporphyrin.
